# The Guild Model of CF Airway Microbial Ecology

**DOI:** 10.1128/mbio.03668-25

**Published:** 2026-04-30

**Authors:** Sage J. B. Dunham, Gregory A. Willkeen, Ben Darby, Jodi M. Corley, Andrea Hahn, Isaac Klapper, Heather D. Bean, Lindsay J. Caverly, Christina S. Thornton, Christian Martin, Robert A. Quinn, Stefanie Widder, Barbara A. Bailey, Brandie D. Wagner, Neha Garg, Paul J. Planet, Ryan C. Hunter, John J. LiPuma, Forest Rohwer, Katrine L. Whiteson

**Affiliations:** 1 Department of Molecular Biology and Biochemistry, University of California Irvine 8788, Irvine, California, USA; 2 Department of Biology, San Diego State University 7117, San Diego, California, USA; 3 Department of Medicine, National Jewish Health 2930, Denver, Colorado, USA; 4 Division of Infectious Diseases, Children's National Hospital 8404, Washington, DC, USA; 5 Center for Precision Medicine and Genomics Research, Children's National Research Institute 580536 , Washington, DC, USA; 6 Department of Pediatrics, George Washington University School of Medicine and Health Sciences 43989, Washington, DC, USA; 7 Department of Mathematics, Temple University 6558, Philadelphia, Pennsylvania, USA; 8 School of Life Sciences, Arizona State University 7864, Tempe, Arizona, USA; 9 Center for Fundamental and Applied Microbiomics, The Biodesign Institute 43363 , Tempe, Arizona, USA; 10 Department of Pediatrics, University of Michigan Medical School 12266 , Ann Arbor, Michigan, USA; 11 Department of Medicine and Microbiology, Immunology and Infectious Diseases, University of Calgary 2129, Calgary, Canada; 12 Centro de Biodiversidad y Descubrimiento de Drogas, Instituto de Investigaciones Cientíﬁcas y Servicios de Alta Tecnología, City of Knowledge, Panama City, Republic of Panama; 13 Sistema Nacional de Investigación, Secretaría Nacional de Ciencia, Tecnología e Innovación, Panama City, Republic of Panama; 14 Department of Biochemistry and Molecular Biology, Michigan State University 3078, East Lansing, Michigan, USA; 15 Department of Medicine 1, Research Division Infection Biology, Medical University of Vienna 27271, Vienna, Austria; 16 Department of Mathematics and Statistics, San Diego State University 7117, San Diego, California, USA; 17 Department of Biostatistics and Informatics, University of Colorado 12226, Denver, Colorado, USA; 18 School of Chemistry and Biochemistry, Georgia Institute of Technology 1372, Atlanta, Georgia, USA; 19 Perelman School of Medicine, University of Pennsylvania 6572, Philadelphia, Pennsylvania, USA; 20 Children’s Hospital of Philadelphia, Philadelphia, Pennsylvania, USA; 21 Department of Microbiology and Immunology, Jacobs School of Medicine and Biomedical Sciences, University at Buffalo 12292 , Buffalo, New York, USA; Kobenhavns Universitet, Copenhagen, Denmark

**Keywords:** cystic fibrosis, microbial ecology, ecological guilds, airway microbiome, host–microbe interactions, polymicrobial infections, ecological modeling

## Abstract

Ecological guilds are groups of organisms that utilize the same class of resources and occupy similar niches, regardless of their taxonomic identities. Here we propose the Guild Model for Cystic Fibrosis Airway Microbial Ecology, which considers the ecological function and wider role of each microbe in the ecosystem. This model consists of four functional guilds: (i) “Brewers” metabolize host-derived substrates (e.g., mucins) and produce fermentation products; (ii) “Drunkards” exploit the metabolic niche built by Brewers, consuming fermentation products and secreting exopolysaccharides to build biofilms; (iii) “Putrifiers” produce toxic compounds causing inflammation and tissue necrosis; and (iv) “Nihilists” are specialist pathogens characterized by intracellular or lytic life cycles and cytotoxin production. By focusing on microbial function and the broader community context, this model offers a refined framework for interpreting cystic fibrosis airway ecology. Although developed for CF, the Guild Model is adaptable to other diseases influenced by microbial ecology.

## OPINION/HYPOTHESIS

In people with cystic fibrosis (pwCF), defective function of the cystic fibrosis transmembrane conductance regulator (CFTR) protein impairs airway mucociliary clearance, creating a nutrient-rich, hypoxic airway environment that supports persistent infection by opportunistic pathogens. Over time, these infections become more entrenched due to age-related changes in the airway environment and the selective pressures imposed by repeated antibiotic exposure. To better understand the ecological dynamics of these polymicrobial infections, researchers have developed conceptual models grounded in ecological theory. For example, the climax–attack model posits a dynamic interplay between stable, well-adapted climax communities and transient, virulent attack communities that emerge during exacerbations of respiratory symptoms (i.e., pulmonary exacerbations) (1). The island biogeography model frames the airways as spatially structured habitats, where microbial infection is shaped by immigration, extinction, and dispersal from upper airway reservoirs (2). Ecological network models have further revealed that microbial interactions—such as antagonism between *Pseudomonas aeruginosa* and anaerobic bacteria—are closely associated with community stability and treatment outcomes (3, 4). System-level approaches that integrate microbial ecology, host physiology, and metabolic interactions have been proposed to help us move beyond taxonomic profiling and toward predictive, mechanistic models of the disease (5). Together, these frameworks have advanced our understanding of the cystic fibrosis (CF) airway microbiome as a complex, individualized, and dynamic ecosystem.

Building on this foundation, we propose a new model based on ecological guilds centered around microbial function in the spirit of Winogradsky, where environmental gradients drive microbial organization (6). Guilds transcend the species and metrics garnered from taxonomic analysis and differ from functional groups by degree of overlap in niche or resource utilization. The guild framework is supported by findings that mucin-degrading anaerobes generate fermentation products (e.g., acetate and propionate) that support the growth of pathogens like *P. aeruginosa*, highlighting the importance of metabolic interdependencies in shaping disease progression (7).

## THE GUILDS

Our understanding of CF airway microbial ecology benefits from considering metabolic function in addition to taxonomy. CF airway communities can be categorized into four broad guilds based on a chain of resource utilization that begins with two key resources: oxygen and mucin. In healthy airways, microbial load is low and microbes have access to an ample supply of mucin and oxygen; mucin is continually secreted by airway epithelial cells, and oxygen-rich air is drawn into the airway through respiration. However, as viscous mucus obstructs airways in the CF lung, both resources become limited (7).

**Fig 1:**
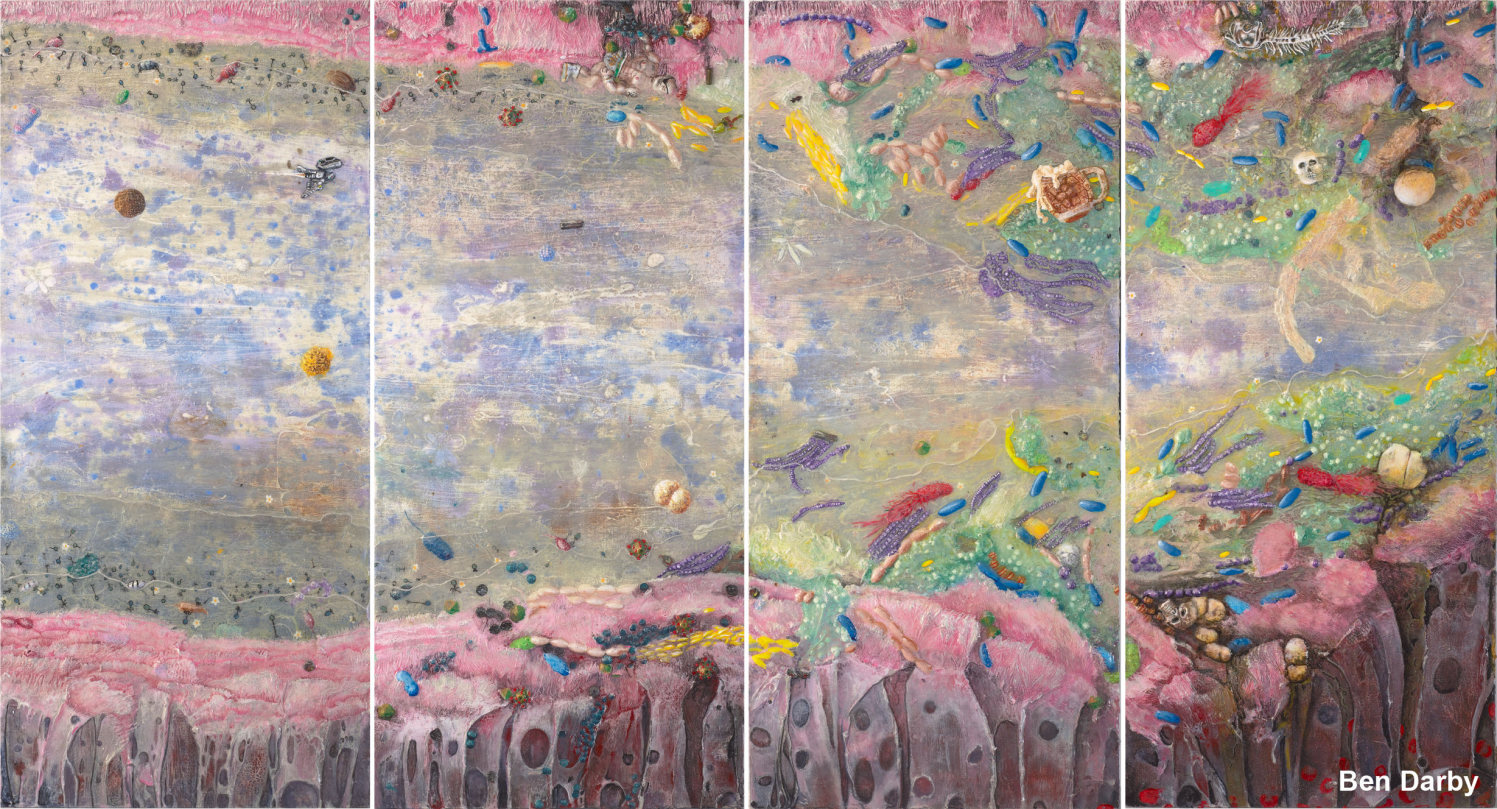
The Guild Model of CF Airway Microbial Ecology represented in a painting of CF airway disease progression. This figure uses guilds, or specific functional categories, of CF pathogens (based on bacterial metabolism) to explain short- and long-term progression of CF airway disease. An interactive version of this painting is available at https://cfguilds.org/ (12). Panel 1: the healthy human airway. A healthy airway lumen with abundant oxygen and mucins flowing freely over epithelial cells. Beating cilia move mucus, which carries debris and microbes with it, out of the lungs. The high turnover rate of mucins (comprising electron donors) and the flow of mucus makes this environment difficult to colonize. The pollen and dinosaur robot represent foreign matter, which is normal in a healthy airway. Panel 2: early CF airway infection. An airway is invaded and infected by microbes, including respiratory viruses and members of the non-CF specific guild, the Nihilists. Infection results in damage to epithelial cells. Abundant oxygen and mucin lead to the emergence of Brewers. The pirate baby in the upper portion of this panel is an allegorical representation of a Nihilist. Panel 3: early CF airway damage. The lung of a person with CF, wherein the mucus is viscous and adherent due to the dysfunctional CFTR. This leads to a low mucin turnover, little to no mucus flow, and reduced oxygen availability in some microenvironments. This lung is being further infected by CF-specific guilds of pathogens, the Brewers and Drunkards. The beer stein in this panel represents the Brewers. Panel 4: advanced CF airway disease. A highly constricted airway containing all three CF-specific guilds: Brewers, Drunkards, and Putrifiers. The low mucus turnover and thick biofilm inhibit efficient oxygen uptake by the host or their resident microbes. Putrifiers produce polyamines such as putrescine and spermidine as a byproduct of their anaerobic metabolism. These polyamines can accumulate to toxic levels and kill the epithelial cells directly or cause inflammation and further neutrophil infiltration. The skulls and fish bones in this panel represent the breakdown of dead cells by Putrifiers.

The chain of resource utilization starts with the first functional group in the model, the “Brewers” (see Fig. 1 and Table 1). Brewers are defined by their ability to metabolize host-derived substrates (e.g., mucins) and make nutrients available for other microbes, either by liberating mucin constituents (e.g., glycans and amino acids) or producing short-chain fatty acids. Examples include eukaryotic opportunistic pathogens such as *Candida* spp., which can cleave mucin chains, freeing sialic acid and fucose for consumption by itself and other microbes (8). Prokaryotic Brewers can be facultatively anaerobic or strictly anaerobic bacteria capable of fermentation, such as *Streptococcus* spp. or *Staphylococcus* spp., that produce nutrients including short-chain fatty acids or lactate as a byproduct of their metabolism (9, 10). Lactate has been reported at elevated levels in CF sputum during pulmonary exacerbations, approximately 400 µM compared to 26 µM during periods of clinical stability (11). Note that the term “Brewers” was intentionally chosen over “Fermenters” to avoid confusion with traditional metabolic classifications in clinical microbiology. While these microbes do engage in fermentation, “Brewers” better conveys their foundational ecological role in producing metabolic byproducts that shape the airway environment for other guilds.

**TABLE 1:** Guild designations for a list of common microorganisms found in the CF airway^*a*^

Guild	Example	Rationale	References
Brewers metabolize host-derived substrates (e.g., mucins) and produce fermentation products.	*Streptococcus* spp.	Facultative anaerobes that ferment carbohydrates; include genes such as als, *budA*, and *budB* for 2,3-butanedione synthesis, which support acidification and cross-feeding with other community members.	Whiteson et al. (13)
	*Gemella* spp.	Fermentative commensals commonly found in oral and upper airway sites can metabolize mucins and carbohydrates, producing lactate and formate.	Torres-Morales et al. (14)
	*Candida* spp.	Ferments sugars under hypoxic conditions to produce ethanol and other organic acids. This process involves genes such as pyruvate decarboxylase (e.g., *PDC1*) and alcohol dehydrogenases family genes.	Chen et al. (15) and Gutiérrez-Corona et al. 16
	*Rothia* spp.	Facultative fermenters that utilize mucin-derived sugars and contribute to lactate and acetate pools, supporting anaerobic cross-feeding, possess lactate dehydrogenase (*LDH*) and other fermentation-related genes.	Gao et al. (17) and Rigauts et al. (18)
	*Lactobacillus* spp.	Classical lactic acid bacteria that ferment host and dietary carbohydrates into lactate, influencing airway pH and microbial community structure. This activity is mediated by LDH glycolytic enzymes that drive pyruvate-to-lactate conversion.	Mejía-Caballero and Marco (19)
	*Staphylococcus* spp.	Capable of mixed-acid fermentation and facultative anaerobic metabolism under low-oxygen airway conditions; well adapted to CF airways, most likely to dominate sputum microbiome, especially early in life	Conrad et al. (1), Widder et al. (4), and Bevivino et al. (5)
	*Prevotella* spp.	Mucin-degrading fermenter producing short-chain fatty acids (including propionate and acetate) involving glycosyl hydrolase and sulfatase genes (e.g., *GH16*)	Flynn et al. (7), Crouch et al. (9), and Silveira et al. (20)
Drunkards exploit the metabolic niche built by Brewers, consuming fermentation products and secreting exopolysaccharides to build biofilms.	*Pseudomonas aeruginosa*	Possesses the alginate biosynthesis operon (*algD*, *algU*, *algA*, etc.), enabling production of exopolysaccharides critical for biofilm formation; also produces Pel and Psl; metabolizes fermentation byproducts such as lactate and ethanol, produced by Brewers, via genes like L-lactate dehydrogenase and ethanol dehydrogenase (*exaA*)	May 1991 (21), Chen 2014 (15), Nguyen 2016 (22), Palmer et al. (23), and Quinn et al. (24)
	*Achromobacter* spp.	*Achromobacter* spp. persist in CF airways by exploiting low-oxygen niches through denitrification and iron acquisition, while pathoadaptive mutations in regulatory genes (*bigR*, *phoQ*, *spoT*, and *cpxA*) enhance biofilm formation and stress tolerance.	Billiot et al. (25) and Khademi et al. (26)
	*Burkholderia* spp.	Capable of producing a variety of exopolysaccharides that contribute to biofilm production and host immune cell evasion, capable of metabolizing lactate using d-lactate dehydrogenase	Whiteson et al. (2), O’Toole et al. (27), and Silva et al. (28)
	*Stenotrophomonas maltophilia*	Possesses genes for lactate dehydrogenase (*LDH*), and multiple virulence factors, including those that exhibit cytotoxic and morphological effects on host cells (*stmPr1* and *stmPr2*), biofilm formation (*smf-1* and *pilU*), and alginate biosynthesis (*spgM*, *rmlA*, and *rpfF*)	Mojica et al. (29)
	*Rothia* spp.	Consumes fermentation products, including lactate (*LDH*) and acetate (acetate kinase); forms biofilms and has EPS synthesis and adhesion-related genes	Lim et al. (30)
	Non-tuberculous mycobacteria	*M. avium* sub sp. *hominissuis* utilizes short-chain fatty acids like propionic and butyric acid to augment metabolic activity across several physiological conditions, including during both planktonic and biofilm-associated growth.	Silva et al. (31)
Putrifiers produce toxic compounds causing local inflammation and tissue necrosis.	*Prevotella* spp.	Aerotolerant anaerobes that induce Th-17 immunity, produce protease, and often require other supportive bacteria in the community	Larsen (32) and Jean-Pierre et al. (33)
	*Veillonella* spp.	*Veillonella* infections increase polyamine concentrations (including putrescine), possibly through metabolic interactions rather than direct synthesis.	Salliss et al. (34)
	*Porphyromonas* spp.	Some species produce gingipains (RgpA, RgpB, and Kgp), cysteine proteases that degrade host proteins, disrupt cytokines, and cause inflammation.	Ciaston et al. (35)
	*Haemophilus* spp.	Some species produce lipooligosaccharides (LOS), which triggers inflammation.	Huska and Ulanova (36)
	*Fusobacterium* spp.	Some species produce putrescine via ornithine decarboxylation, mediated by the *oda* gene. Some species express adhesins, such as Fap2 and CbpF, which bind host receptors and facilitate immune evasion and inflammation.	Xu et al. (37), Schöpf et al. (38), and Shen et al. (39)
Nihilists are specialist pathogens characterized by intracellular or lytic life cycles and cytotoxin production.	*Gemella haemolysans*	Exhibits virulence through mucin-binding protein (MucBP)-facilitated surface adhesins and choline-binding protein-facilitated epithelial interaction	Shumyatsky et al. (40) and García López and Martín-Galiano (41)
	*Streptococcus pyogenes*	Group A streptococcus can invade and induce apoptosis in epithelial cells via type 6 M protein (M6) and fibronectin-binding F1 (F1) protein. Apoptosis is mediated by production of reactive oxygen species.	Nakagawa et al. (42), Aikawa et al. (43), and Tsai et al. (44)
	Some *Staphylococcus* spp.	Can induce apoptosis in pulmonary epithelial cells via cytoplasmic and mitochondrial Ca^2+^ overload; mediated by calcium signaling, “one carbon pool by folate,” and “folate biosynthesis” pathways	Stelzner et al. (45)
	*Haemophilus influenzae*	Persists in CF airways by invading epithelial cells and forming intraepithelial reservoirs. High-molecular-weight adhesins HMW1 and HMW2 facilitate adherence and internalization. IgA1 proteases (encoded by *igaA* and *igaB*) promote intracellular persistence through cleavage of host LAMP1 and immune evasion. LOS and autotransporter proteins such as Hap further contribute to epithelial damage and immune modulation, enabling long-term colonization.	Goyal et al. (46) and Clementi et al. (47)
	SARS-CoV-2	Induces programmed cell death to clear viral infection	Shao et al. (48)
	Influenza	Induces programmed cell death to clear viral infection	Shao et al. (48)
	Respiratory syncytial virus	Induces programmed cell death to clear viral infection	Shao et al. (48)

^*a*^ This table provides example guild designations that can be expanded and modified to fit the needs of the individual researcher. The assignment of each microbe to a guild was made based on the personal experiences of the authors and a review of literature. The rationale underpinning each assignment includes the capacity of the microbe to form specific functional roles indicative of a given guild, which are encoded in their genome and expressed in the proteins and metabolites that they consume, produce, or induce. Please note that the guild membership of a given microbe can be both temporally and spatially dynamic. Microbes can reside in one or more guilds, depending on local conditions.

A key feature of the Brewers is that they make energy available for the second guild of microorganisms, the “Drunkards,” which are biofilm-producing bacterial genera such as *Pseudomonas*, *Achromobacter*, *Burkholderia*, and *Stenotrophomonas.* Drunkards exploit the metabolic niche built by the Brewers and in turn build another niche, i.e., a biofilm, by secreting alginate and/or other exopolysaccharides, facilitating the aggregation of microbes into a complex matrix (21). Drunkards may also respond to Brewers’ fermentation products (2,3-butanediol or ethanol [13, 15, 20, 22]) by forming biofilms, which can lead to increased infection of the airways as well as persistence through antibiotic treatments.

Once the accumulated metabolism of the Brewers and Drunkards depletes enough oxygen and lowers the pH, the next functional group, the “Putrifiers,” can proliferate. Putrifiers are bacteria characterized by anaerobic metabolism, such as *Prevotella* and *Veillonella*. Anaerobic metabolism by Putrifiers results in the production of toxic compounds such as putrescine and spermidine, which can cause local inflammation and necrosis (49). Putrescine has been measured at between 100 and 600 µM in CF sputum around the time of pulmonary exacerbation, which is approximately an order of magnitude greater than what is reported during periods of clinical stability (approximately 25 µM) (11, 50). This interplay between Brewers, Drunkards, and Putrifiers results in the progressive lung damage and loss of lung function that characterizes CF (Fig. 1).

The final guild is a group of stand-alone pathogens, the “Nihilists,” that do not partake in the chain of resource utilization. This group is a loose collection of viruses and bacteria that are independently cytotoxic, cytolytic, or carry out intracellular life cycles. Nihilists include adenoviruses, which lyse human cells as a part of their life cycle, or bacteria such as *Streptococcus pyogenes*, which produce hemolysins that rupture red blood cells. This cohort of Nihilists is thought to be capable of instigating inflammation and lung damage in pwCF without the need to engage with the rest of the guilds present in the CF airway. These pathogens can impact the airway at any stage of CF disease, often triggering or worsening pulmonary exacerbations.

Guild membership of a given microbe can be dynamic, both temporally and spatially. It is perhaps helpful to conceptualize guilds as a Venn diagram, with some members residing in one or more guilds, depending on local conditions. This flexibility introduces functional redundancy into the system, which enhances ecological stability by buffering against the loss of individual species. Ecological theory and microbiome studies suggest that systems with overlapping functional roles tend to be more resilient to perturbations, as they preserve critical ecosystem functions even when community composition shifts (51, 52). In chronic CF infection, this dynamic nature may also be shaped by genetic adaptation, as microbes evolve in response to selective pressures such as host immunity, antibiotic exposure, and nutrient availability. These ecoevolutionary feedbacks can further influence guild structure and function, reinforcing the resilience of the microbial community over time (53).

The Guild Model refines earlier frameworks like the climax–attack model, which posits that there are two distinct communities of bacteria in the CF airway (1). The climax community is composed of well-adapted, long-term residents such as *P. aeruginosa* and strict anaerobes, while the attack community includes transient maladapted microbes associated with inflammation, such as *Haemophilus influenzae* and *Streptococcus* spp. (54, 55). Rather than including every permutation of these microbial guilds’ actions and interactions, we propose the Guild Model as a shift in thinking about the establishment and succession of CF airway infection, pulmonary exacerbation, and progressive lung disease. By grouping microbes into functional guilds, researchers can reduce the noise and dimensionality of their data sets and better interpret the ecological dynamics at play.

## INCORPORATING EXISTING DATA SETS INTO THE GUILD FRAMEWORK

**Fig 2:**
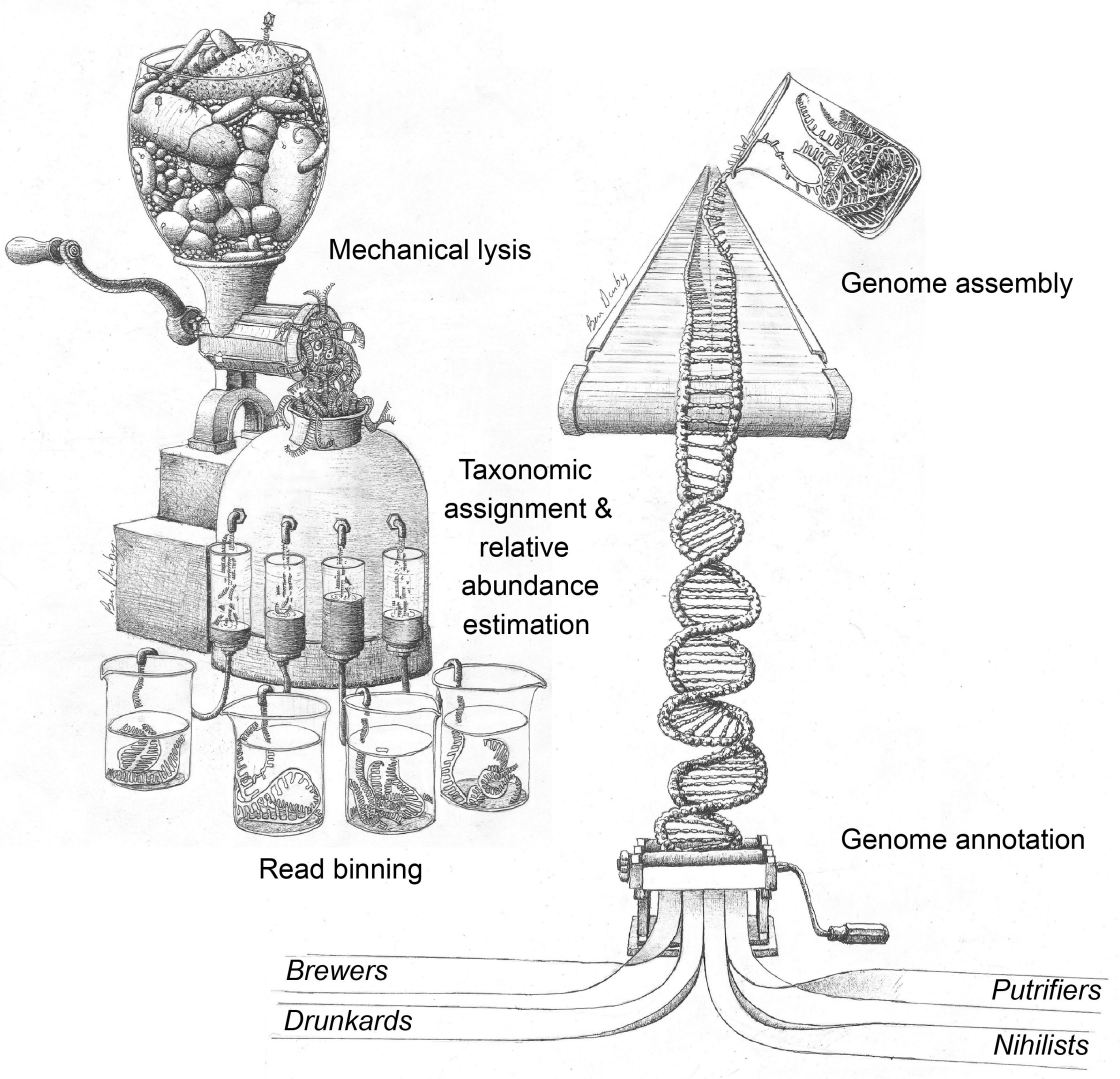
Workflow for binning microbes into the four functional categories proposed by the Guild Model of CF Airway Microbial Ecology. The process begins at the top left, where the sausage grinder represents the process of lysing all bacteria, fungi, and viruses to extract and sequence the DNA. Whole-genome shotgun reads are processed and assigned to taxonomic units based on their similarity to database sequences of known taxa. While the figure depicts a whole-genome approach for illustrative purposes, the workflow is modular and can accommodate alternative strategies such as marker gene-based profiling (e.g., 16S rRNA for bacteria, mOTUs for prokaryotes, SingleM for bacteria and archaea, ITS1 for fungi, and geNomad for taxon-specific viral markers) and targeted databases (e.g., 16S, mOTUs, and SingleM) for bacteria and archaea, and targeted databases for fungi and viruses. The relative abundance of each species is estimated by counting reads and normalizing to genome size, as depicted by the short pieces of DNA accumulating in the containers. Binned reads are assembled into contigs using their nearest relative as a scaffold. Once assembled, the genome is annotated. Each microbe is assigned to a guild according to the predicted functions of genes present in its genome. When available, metatranscriptomes, proteomes, or metabolomes could be used to improve guild classification by confirming the expression of key genes or the production of metabolic byproducts (not shown).

To build out this concept, a workflow is proposed in Fig. 2 that outlines the analysis of sputum metagenomes and the assignment to functional guilds based on gene content. The genome encodes the metabolic and pathogenic capabilities of the organism, and its gene content can be used to classify the organism as a Brewer, Drunkard, Putrifier, or Nihilist. The figure illustrates a whole-genome shotgun (WGS) sequencing approach; however, the framework is adaptable to alternative strategies, including marker gene-based profiling (e.g., 16S rRNA for bacteria, mOTUs for prokaryotes, SingleM for bacteria and archaea, ITS1 for fungi, and geNomad for taxon-specific viral markers) and targeted databases. Regardless of the method, the goal is to infer gene content and metabolic potential. When available, metatranscriptomes, proteomes, or metabolomes can improve guild classification by confirming the expression of key genes or the production of metabolic byproducts (56).

WGS sequencing provides broader coverage and enables functional inference; however, quantifying microbial abundance across bacteria, fungi, and viruses presents several challenges. Such an analysis would require large, domain-specific reference databases and substantial computational resources. Additionally, differences in genome size, GC content, and copy number variation (e.g., rRNA operons in bacteria and segmented genomes in viruses) complicate normalization and abundance estimation across domains (57). Viral genomes, in particular, are highly diverse and underrepresented in reference databases, making detection and quantification difficult (58). Fungal genomes are larger and more complex, often requiring specialized assembly and annotation pipelines. Marker gene-based approaches are efficient and well established for profiling bacteria and archaea, but they typically exclude fungi and viruses and typically offer limited taxonomic resolution, often only to the genus level. Additionally, functional inference from marker genes is indirect and frequently inaccurate without consideration of full genomic context, which constrains their utility for comprehensive ecological modeling.

Our framework aims to capture the full breadth of microbial diversity in the CF airway, including bacteria, fungi, and viruses, because each domain meaningfully contributes to community structure and host interactions. For example, fungal pathogens such as *Aspergillus fumigatus* and viruses like bacteriophages can modulate bacterial behavior and immune responses, making their inclusion biologically meaningful. It is also important to consider mobile genetic elements such as prophages and plasmids, which are known to carry genes for virulence factors (e.g., exotoxins, immune evasion, and antibiotic resistance) that allow any Brewer, Drunkard, or Putrifier to become a Nihilist. For example, an otherwise benign strain of *Escherichia coli* could become cytotoxic via Shiga toxin-encoding prophages from the O157:H7 serogroup (59). While WGS sequencing and consideration of all domains may not be strictly necessary for every application, we argue that doing so enhances ecological resolution and hypothesis generation, particularly in polymicrobial environments like the CF airway.

## NOVEL EXPERIMENTAL MODELS

Effective modeling of CF airway ecology remains a challenge. While some animal models recapitulate certain hallmarks of CF lung disease (e.g., ferrets and pigs), they do not survive long enough for long-term investigations, and their cost limits large, statistically meaningful studies (60). These constraints have led to the development of synthetic CF sputum media and *in vitro* models, such as the WinCF model (23, 24). Although critical to our understanding, *in vitro* models have shortcomings such as not being chemostats, not incorporating host immune cells, and employing an imperfect substitute for CF sputum as the medium (24). It is also worth noting that increasingly sophisticated metabolic models raise the possibility of personalized *in silico* modeling of the CF airway together with its resident microbiota (27).

The Guild Model offers a conceptual framework for developing and refining these experimental systems. By organizing microbes into functional guilds based on their metabolic roles—such as mucin degradation, fermentation, biofilm formation, or cytotoxicity—researchers can design models that more accurately simulate the ecological succession and metabolic interactions observed *in vivo*. For example, incorporating representative members of the Brewers and Drunkards into artificial sputum media could help recapitulate early-stage biofilm formation and fermentation-driven oxygen depletion. Similarly, introducing Putrifiers under anaerobic conditions could simulate the inflammatory and necrotic environment of advanced disease. Even Nihilists, though often studied in isolation, could be modeled in the context of their interactions with other guilds to better understand their role in exacerbations.

Ultimately, experimental models (whether *in vitro*, *in vivo*, or *in silico*) need to be validated against clinical samples from pwCF. Quantitative validation of a model can be achieved by comparing genomic, transcriptomic, and metabolomic profiles to those derived from CF sputum (61). The Guild Model can serve as a bridge between clinical data and experimental design, helping to ensure that models reflect not just microbial composition but also ecological function and disease relevance.

## BROADER APPLICATION OF THE GUILD MODEL

In presenting the Guild Model of CF Airway Microbial Ecology, we provide a framework for the incorporation of microbial function into our understanding of CF airway infections. Despite our focus on CF, we recognize that the concept of microbial guilds is more broadly relevant to other diseases characterized by microbial dysbiosis and host–microbe interactions.

For example, in ulcerative colitis and Crohn’s disease (two prominent forms of inflammatory bowel disease [IBD]), Brewers such as *Bacteroides* spp. metabolize the intestinal mucus layer of the gastrointestinal tract, producing short-chain fatty acids. The presence of fermentation products supports colonization by biofilm-forming Drunkards, such as adherent-invasive *E. coli* (62). Putrifiers, such as the sulfate-reducing bacteria *Desulfovibrio* spp., generate hydrogen sulfide, a toxic metabolite linked to mucosal inflammation and epithelial damage in ulcerative colitis (63). Acting as a Nihilist, *Clostridium perfringens* can independently exacerbate intestinal inflammation through the secretion of potent cytotoxins that disrupt epithelial integrity, promote immune activation, and contribute to acute tissue damage. *C. perfringens* often acts opportunistically during IBD flares or following antibiotic use, without requiring support from the broader microbial community (64).

In chronic wounds, such as diabetic foot ulcers, microbial consortia often include fermentative anaerobes, biofilm producers, cytotoxic pathogens, and proteolytic anaerobes, paralleling the Brewers, Drunkards, Nihilists, and Putrifiers of our model. Brewers, such as *Peptostreptococcus* spp., ferment host-derived amino acids, peptides, and necrotic tissue proteins, establishing a low-oxygen, nutrient-rich environment. Drunkards, including *P. aeruginosa*, exploit these conditions to form resilient biofilms that resist immune clearance and antibiotic treatment. Putrifiers, such as *Prevotella* spp., contribute to tissue degradation and inflammation through the production of proteases and other virulence factors that break down host proteins (65).

By focusing on ecological function rather than taxonomy alone, the Guild Model offers a flexible conceptual tool for dissecting microbial ecosystems across diverse anatomical sites and disease processes. This approach may facilitate the development of ecologically informed diagnostics and therapeutics, such as interventions that disrupt harmful guild interactions or promote beneficial ones.

## CONCLUSION

Researchers and care providers can exploit the principles of microbial ecology to better understand and manage airway infections in CF, which persist even with highly effective CFTR modulator therapies (66). At a basic biology level, our understanding of CF airway microbial communities would benefit from moving beyond classical taxonomic approaches toward incorporating functional guild-centric analyses. Applied properly, the guild framework can help reduce the noise and dimensionality of the data by focusing on the key functions of each microbe. Designing experiments based on this guilds concept has the potential to better define microbial community dynamics, advance our appreciation of airway infection pathophysiology, and identify ecological tipping points and novel treatment strategies. Such models would enable controlled, hypothesis-driven assessment of microbial succession, resilience, and recovery, particularly in the context of therapeutic interventions. Ultimately, the Guild Model aims to bridge ecological theory and clinical application, supporting the development of more targeted and effective interventions for pwCF.
